# Chest CT Findings at Six Months Following COVID-19 ARDS – Correlation With the mMRC Dyspnea Scale and Pulmonary Function Tests

**DOI:** 10.3389/bjbs.2024.12871

**Published:** 2024-07-10

**Authors:** Mandeep Garg, Nidhi Prabhakar, Shritik Devkota, Sahajal Dhooria, Uma Debi, Ashish Dua, Tarvinder Singh, Muniraju Malarakunte, Harsimran Bhatia, Manavjit Singh Sandhu

**Affiliations:** ^1^ Department of Radiodiagnosis and Imaging, Post Graduate Institute of Medical Education and Research, Chandigarh, India; ^2^ Department of Pulmonary Medicine, Post Graduate Institute of Medical Education and Research, Chandigarh, India

**Keywords:** COVID-19 ARDS, post-severe COVID-19, long Covid, CT chest-mMRC dyspnoea scale association, CT chest-PFT association

## Abstract

**Background:** Many survivors of severe COVID-19 pneumonia experience lingering respiratory issues. There is limited research on follow-up chest imaging findings in patients with COVID-19 ARDS, particularly in relation to their mMRC dyspnea scores and pulmonary function tests (PFTs). This study addresses this gap by investigating the clinical characteristics, mMRC dyspnea scores, PFTs, and chest CT findings of COVID-19 ARDS patients at the 6 months post-recovery. By analyzing these variables together, we aim to gain a better understanding of the long-term health consequences of COVID-19 ARDS.

**Methods:** This prospective observational study included 56 subjects with COVID-19 ARDS with dyspnea at the six-month follow-up visits. These patients were evaluated by chest CT, mMRC dyspnea scale, and PFT. The CT severity score was calculated individually for each of the four major imaging findings - ground glass opacities (GGOs), parenchymal/atelectatic bands, reticulations/septal thickening, and consolidation - using a modified CT severity scoring system. Statistics were carried out to find any association between individual CT chest findings and the mMRC dyspnea scale and forced vital capacity (FVC). p values < 0.05 were considered statistically significant.

**Results:** Our study population had a mean age of 55.86 ± 9.60 years, with 44 (78.6%) being men. Grades 1, 2, 3, and 4 on the mMRC dyspnea scale were seen in 57.1%, 30.4%, 10.7%, and 1.8% of patients respectively. Common CT findings observed were GGOs (94.6%), reticulations/septal thickening (96.4%), parenchymal/atelectatic bands (92.8%), and consolidation (14.3%). The mean modified CT severity scores for GGOs, reticulations/septal thickening, parenchymal/atelectatic bands, and consolidation were 10.32 ± 5.51 (range: 0–21), 7.66 ± 4.33 (range: 0–19), 4.77 ± 3.03 (range: 0–14) and 0.29 ± 0.91 (range 0–5) respectively. Reticulations/septal thickening (p = 0.0129) and parenchymal/atelectatic bands (p = 0.0453) were associated with an increased mMRC dyspnea scale. Parenchymal/atelectatic bands were also associated with abnormal FVC (<80%) (p = 0.0233).

**Conclusion:** Six-month follow-up chest CTs of COVID-19 ARDS survivors with persistent respiratory problems showed a statistically significant relationship between increased mMRC dyspnea score and imaging patterns of reticulations/septal thickening and parenchymal/atelectatic bands; while parenchymal/atelectatic bands also showed a statistically significant correlation with reduced FVC.

## Introduction

The impact of the COVID-19 pandemic has transcended the initial phase of infection, with mounting scientific and clinical evidence of persistent illness in a significant number of patients following recovery from acute illness [1–4]. Frequently observed long-term effects of COVID-19 include fatigue, shortness of breath, cough, chest pain, joint pain, and cognitive difficulties [3–5]. Such a diverse array of long-term health sequelae has been given many names such as “Long COVID,” “Chronic COVID,” “Ongoing Symptomatic COVID-19,” “Post-Acute COVID-19 Syndrome,” “Post-Acute Sequelae of COVID-19” and “Post-COVID-19 condition” [3–7]. While a unified nomenclature and definition to describe this condition remain elusive, the term “Long COVID” has garnered increasing traction within the medical community [3, 4, 6, 7].

Chest computed tomography (CT) has played a critical role in the evaluation of lung changes in COVID-19 survivors [3, 4, 8–10]. The burden of Long COVID is enormous, with persistent lung parenchymal abnormalities seen in up to one-third of COVID-19 hospitalized patients, as reported in recent research and meta-analyses [11, 12]. Ground-glass opacities (GGOs), septal thickening, parenchymal/atelectatic bands, and traction bronchiectasis are the most common radiological abnormalities seen in these patients [1, 2, 10–15].

However, studies specifically investigating the long-term outcomes of patients with COVID-19 acute respiratory distress syndrome (ARDS) are limited. Our present study evaluated clinical features, pulmonary function tests (PFTs), and lung parenchymal abnormalities on chest CT at the six-month follow-up of Long COVID patients who had developed COVID-19 ARDS during the acute phase. Additionally, we investigated potential associations between these CT findings and the modified Medical Research Council (mMRC) dyspnea scale and forced vital capacity (FVC).

## Materials and Methods

### Study Population

**FIGURE 1:**
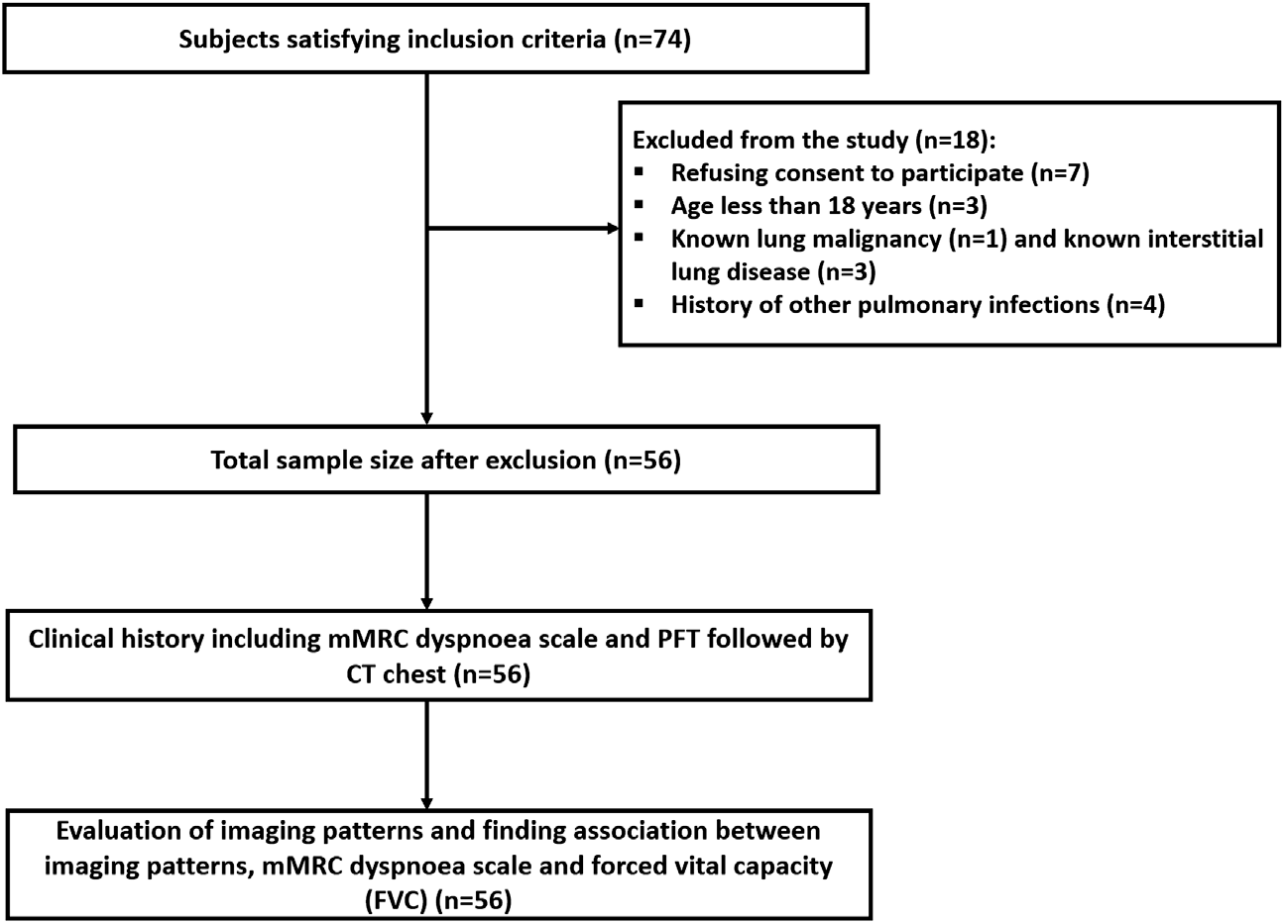
Flowchart depicting the study design and subject process of the study participants.

This was a single-center, prospective observational study approved by the institutional ethics committee (IEC number - INT/IEC/2021/SPL-288) and carried out from December 2021 to August 2022. A total of 74 participants who developed acute-phase COVID-19 ARDS, and had persistent respiratory symptoms in the form of dyspnea (shortness of breath) 6 months after discharge, were screened for inclusion. The Berlin criteria for ARDS were taken into consideration for inclusion [16]. Patients with a known history of other pulmonary co-morbidities (pulmonary tuberculosis, pulmonary mucormycosis, pulmonary aspergillosis, any superimposed infection during the acute phase, cystic or interstitial lung disease, and known malignancies), aged less than 18 and who refused to participate in the study were excluded. A total of 56 participants were finally enrolled in the study after exclusion (Figure 1). At the six-month follow-up, participants were evaluated for dyspnea according to the mMRC dyspnea scale [17], PFTs, and abnormalities seen on the chest CT. FVC values obtained from PFT were graded 0 for FVC ≥80% of total lung capacity (TLC) and 1 for FVC <80% of TLC.

The mMRC dyspnea scale [17] is a quick and easy way to measure shortness of breath (dyspnea). As the score increases, breathlessness worsens: grade 0, breathless only with strenuous exertion; grade 1, slightly breathless when hurrying on flat ground or walking uphill; grade 2, walks slower than others due to shortness of breath or needs to stop to catch breath when walking at a normal pace; grade 3, stops to catch breath after about 100 yards (91 m) or a few minutes of walking on flat ground; grade 4, too breathless to leave the house or even to dress or undress. Higher scores on the mMRC scale indicate a greater limitation of daily activities due to shortness of breath.

### Chest CT Acquisition Protocol and Interpretation

All the participants underwent a non-contrast chest CT on a 256-slice CT scanner (Philips Brilliance-iCT256; Koninklijke Philips N.V., Netherlands). Scanning was performed under deep inspiration, from the level of the thoracic inlet to the domes of the diaphragm in helical mode with a fixed tube voltage of 120 kV and automatic exposure control (AEC) modulated tube current. The images were then reconstructed from the helical dataset into 1 mm sections in 1 mm increments using the iDose level 6 hybrid iterative reconstruction software.

All scans were randomly assigned and then independently assessed by two chest radiologists (MG and NP) with 25 and 15 years of experience respectively, who were blinded to the patients’ clinical and laboratory data. Disagreements were resolved through discussion and mutual consensus. Each imaging finding was entered in the data sheet.

We implemented a novel, modified CT severity scoring system (Table 1), building upon the original scoring system by Pan et al. [18]. Lung involvement was assessed on a lobar basis, employing a 0–25 point scale; however, an independent score was given to each radiological finding. We scored the four most prevalent CT imaging findings seen in these patients - GGOs, consolidation, parenchymal/atelectatic bands, and reticulations/septal thickening. Additionally, traction bronchiectasis was assigned a binary score of 0 (absent) or 1 (present). These radiological terms were used according to the current recommendations of the Fleischner Society glossary for accurate and precise imaging descriptions of the imaging patterns [19].

**TABLE 1:** Modified CT severity scoring system used in our study.

Modified CT severity scoring system	
Extent of lobar involvement for each radiological finding	Score
No involvement	0
<5% involvement	1
5%–25% involvement	2
26%–50% involvement	3
51%–75% involvement	4
>75% involvement	5

Total score of each lobe for each finding = 0–5. Total score of 5 lobes for each finding = 0 (no involvement) – 25 (maximum involvement).

### Statistical Analysis

Data were entered into a Microsoft Excel spreadsheet. IBM SPSS Statistics Version 22.0 was used for the analysis. Categorical data were presented as counts and percentages. The mean and Standard Deviation were used to depict normally distributed data. The Wilcoxon signed rank test and the Kruskal-Wallis rank test were used to compare various variables. Fischer’s Exact test was used for count data (traction bronchiectasis). Statistical significance was defined as a *p*-value of less than 0.05.

## Results

The mean age of our study population was 55.86 ± 9.60 years (mean ± SD) with 44 (78.6%) patients being male. Cough, fatigue, and myalgia were present in 71.4%, 46.4%, and 16% of the participants, respectively, at the six-month follow-up visit. The most frequent comorbidity in the study cohort was diabetes (53.5%). Only one participant (1.8%) had class 4 dyspnea, while 57.1%, 30.4%, and 10.7% of the subjects had grade 1, 2, and 3 dyspnea respectively as per the mMRC dyspnea scale. PFTs revealed an FVC of <80% in 36 (64.3%) subjects. Patient demographics and clinical characteristics are elaborated in Tables 2, 3.

**TABLE 2:** Patient demographics (n = 56).

Demographics	
Age (years)	55.08 ± 12.9 (mean ± SD)
**Sex**	**n (%)**
Male subjects	44 (79%)
Female subjects	12 (22%)

**TABLE 3:** Clinical details, mMRC dyspnea scale, and FVC values of the study participants (n = 56).

Patient characteristics	n (%)
Clinical symptoms	
Cough	40 (71.4%)
Dyspnea	56 (100%)
Myalgia	9 (16%)
Fatigue	26 (46.4%)
Clinical history	
Diabetes	30 (53.5%)
Hypertension	22 (39.3%)
Coronary artery disease	6 (10.7%)
Obesity	18 (32.1%)
Smoker	13 (23.2%)
mMRC scale	
0	0
1	32 (57.1%)
2	17 (30.4%)
3	6 (10.7%)
4	1 (1.8%)
FVC	
≥80% of TLC	20 (35.7%)
<80% of TLC	36 (64.3%)

Abbreviations: mMRC, modified Medical Research Council; FVC, forced vital capacity; TLC, total lung capacity.

**FIGURE 2:**
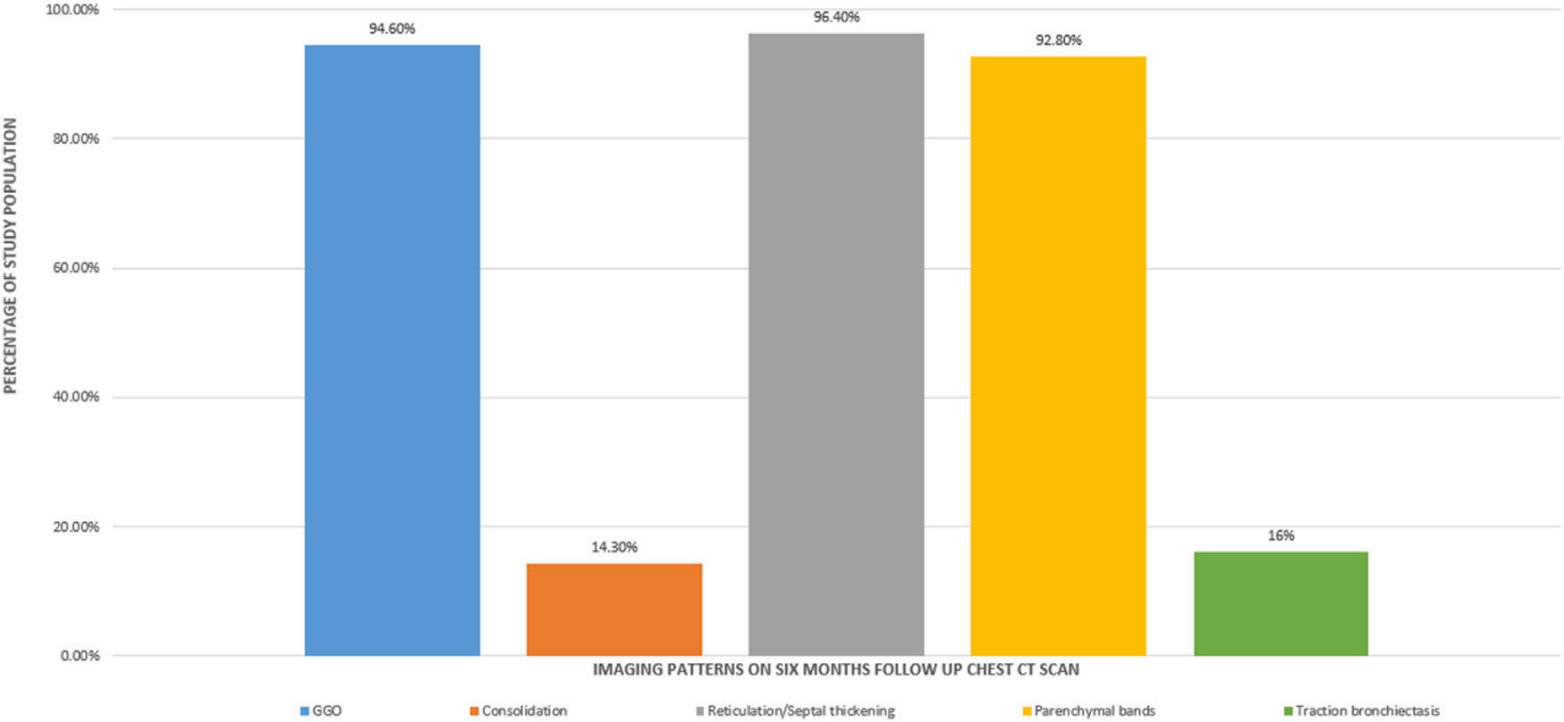
Bar diagram showing the imaging patterns observed at the six-month follow-up CT scan in patients recovering from COVID-19 ARDS.

**FIGURE 3:**
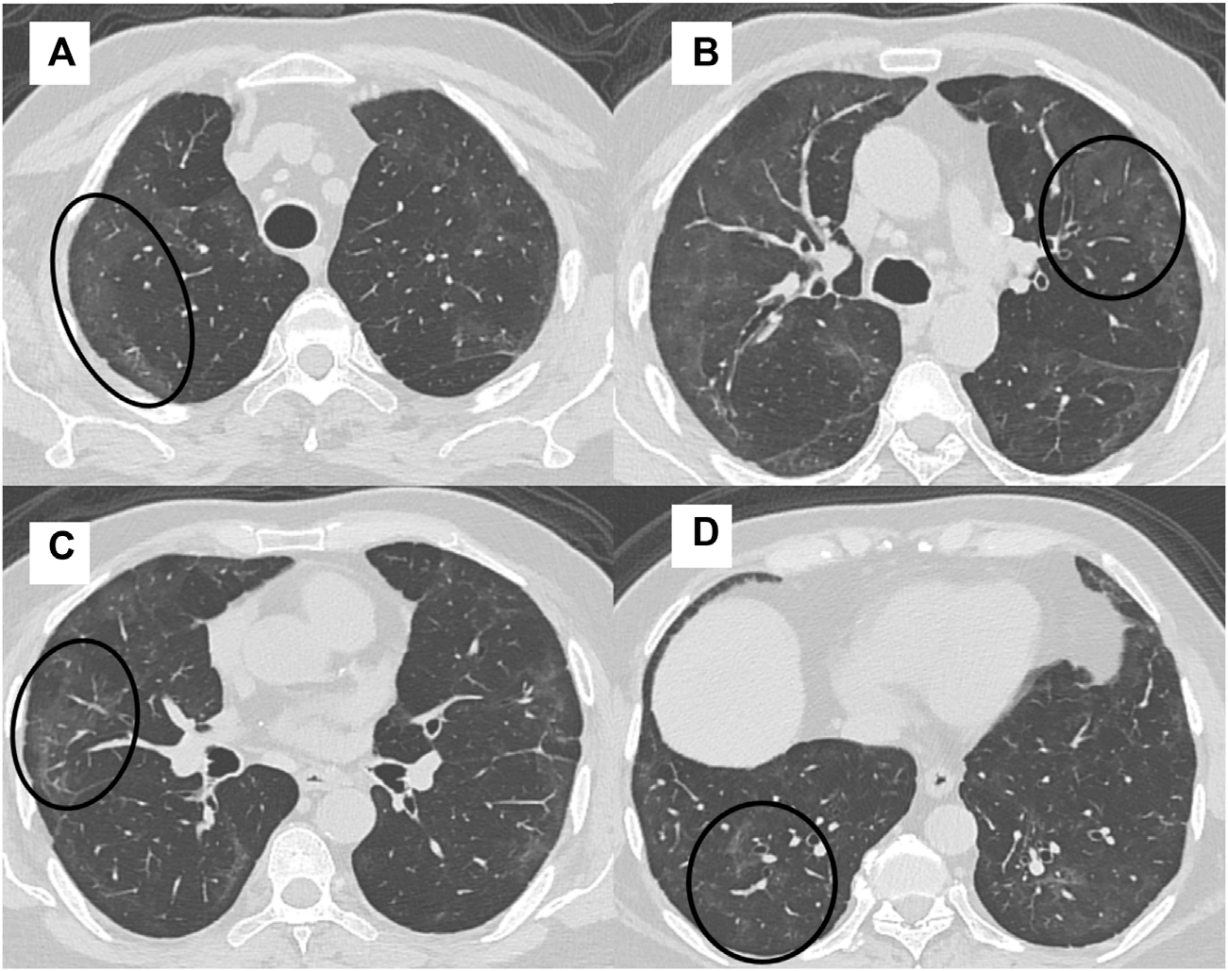
A 41-year-old man with intermittent cough, fatigue, and mMRC class 1 dyspnea had an FVC of 78% at 6 month follow up after COVID-19 ARDS. High-resolution CT (HRCT) chest axial sections **(A–D)** showed multifocal areas of GGOs in both lungs (black circles).

**FIGURE 4:**
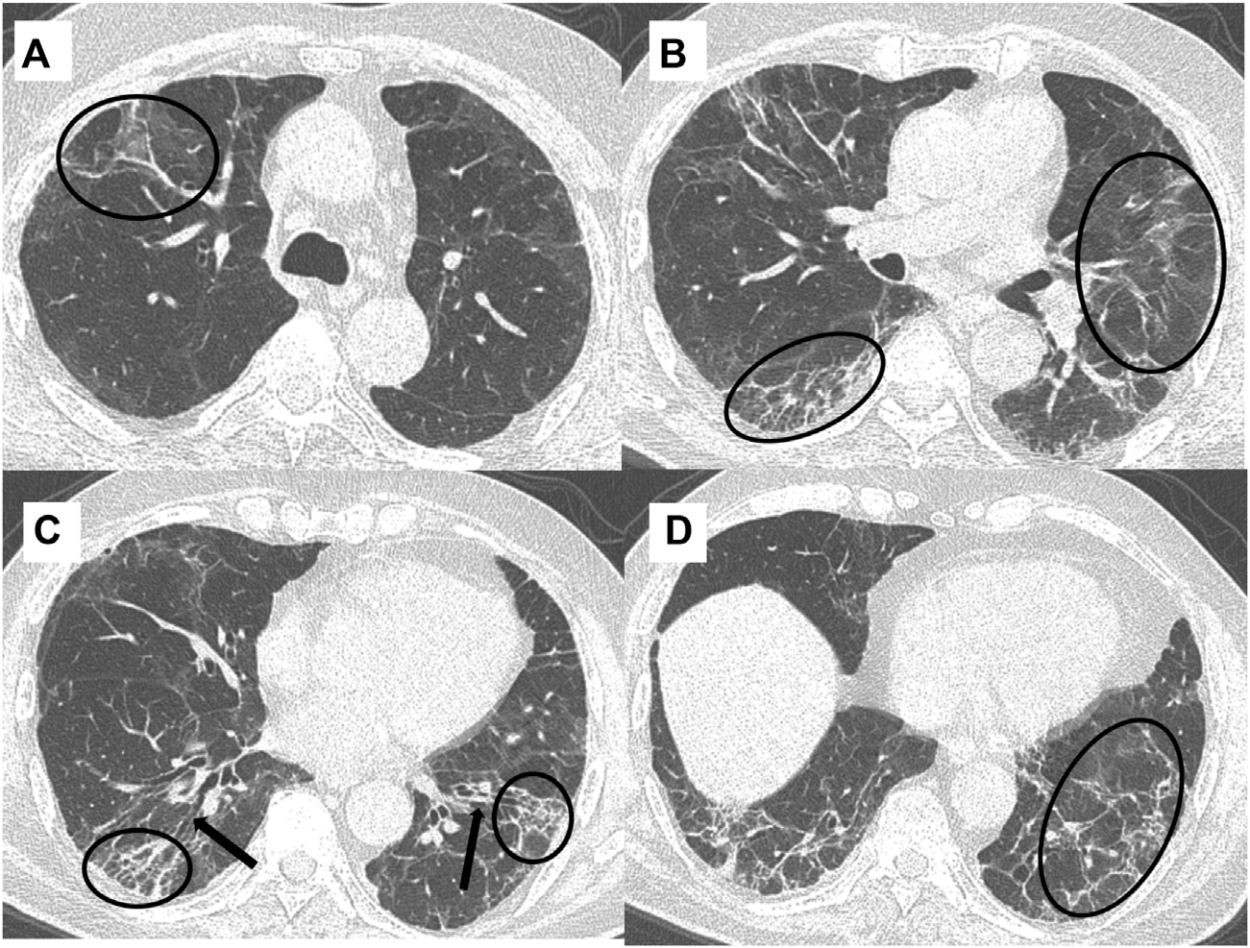
A 49-year-old, COVID-19 ARDS recovered male patient presented with persistent cough and mMRC class 3 dyspnea at six-month follow-up. Spirometry showed an FVC of 60% and high-resolution chest CT (HRCT) axial sections **(A–D)** showed the presence of reticulations/septal thickening and parenchymal bands (black circles), and traction bronchiectasis (black arrows) interspersed with patchy areas of background GGOs.

The mean time between the CT scan and patient discharge was 206.5 ± 28.8 days. The most common imaging patterns seen at six-month follow-up were reticulations/septal thickening (96.4%), GGOs (94.6%), parenchymal bands (92.8%), traction bronchiectasis (16%), and consolidation (14.3%). This is shown in the bar diagram in Figure 2. There was considerable overlap in the imaging patterns, with two or more of these patterns seen within a single lobe in many patients. Figures 3, 4 illustrate the lung parenchymal abnormalities identified on chest CTs done at six-month follow-up in our study cohort. The mean modified CT severity score for GGOs was 10.32 ± 5.51 (range 0–21), followed by 7.66 ± 4.33 (range 0–19) for reticulations/septal thickening, 4.77 ± 3.03 (range 0–14) for parenchymal/atelectatic bands and 0.29 ± 0.91 (range 0–5) for consolidation.

### Association Between CT Findings and Dyspnea Scores at Six-Month Follow-Up

The association between common CT findings including GGOs, reticulations/septal thickening, parenchymal/atelectatic bands, consolidation, and traction bronchiectasis and the mMRC dyspnea scale was evaluated for statistical significance and is summarized in Table 4. With increasing mean CT severity scores of reticulations/septal thickening and parenchymal/atelectatic bands, there were increasing grades of dyspnea on the mMRC scale (from 1 to 3), and these associations were statistically significant (*p*-value < 0.05). However, the association between traction bronchiectasis, GGOs, and consolidation with dyspnea scores was not significant.

**TABLE 4:** Test of association between imaging patterns and the mMRC dyspnea scale.

Variable		mMRC dyspnea scale					*p*-value
		0	1	2	3	4	
Mean modified CT severity score (out of a total of 25)	GGO	0	9.7	10.4	15.0	0	0.0683
	Reticulations/septal thickening	0	7.1	7.4	12.7	0	0.0129
	Parenchymal/atelectatic bands	0	4.5	4.1	8.3	3.0	0.0453
	Consolidation	0	0.41	0.18	0	0	0.8999
Traction bronchiectasis (number of patients)		0	5 (55.56%)	3 (33.33%)	1 (11.11%)	0 (0%)	1.000

Abbreviations: mMRC, modified Medical Research Council; GGO, ground glass opacity.

### Association Between CT Findings and PFTs at Six-Month Follow-Up

The association between CT findings and FVC was also evaluated for statistical significance. In patients with abnormal FVC values (<80%), the mean CT severity score of parenchymal/atelectatic bands was found to be increasing and this association was statistically significant (*p*-value < 0.024). However, there was no significant association seen between FVC and traction bronchiectasis, GGOs, consolidation, or reticulations/septal thickening (Table 5).

**TABLE 5:** Test of association between imaging patterns and the FVC values.

Variable		FVC		*p*-value
		≥80%	<80%	
Mean Modified CT severity score (out of a total of 25)	GGO	8.5	11.3	0.0648
	Reticulations/septal thickening	6.2	8.5	0.0129
	Parenchymal/atelectatic bands	3.6	5.4	0.0453
	Consolidation	0.15	0.36	0.6174
Traction bronchiectasis (number of patients)		3 (33.33%)	6 (66.67%)	1.0000

Abbreviations: FVC, forced vital capacity; GGO, ground glass opacity.

## Discussion

A significant number of COVID-19 survivors continue to experience long-term health problems such as myalgia, fatigue, dyspnea, and cough with some individuals facing even more debilitating organ injuries [3, 4, 20–22]. Such patients often require close clinical, laboratory, and imaging follow-up to monitor the disease progression and identify potential complications. Several studies have shown that chest CT is a valuable imaging tool for assessing lung damage in patients with persistent respiratory symptoms after COVID-19 [1, 3, 11, 13, 20–22].

According to our prospective study, fatigue followed by dyspnea and cough were the most common symptoms at six-month follow-up in our cohort. This is in agreement with the study conducted by Vijaykumar et al [20] who reported dyspnea and cough as the most common respiratory complaints.

The most common CT findings were GGOs, reticulations/septal thickening, and parenchymal/atelectatic bands; observed in more than 90% of participants in our patient cohort. In contrast in a study by Han and Fan et al [23], fibrotic-like lung changes were observed only in 35% of the patients at he six-month follow-up. This discrepancy may be due to the fact that our study group included only subjects who had developed severe disease in the form of ARDS in the acute phase of the illness. On the other hand, a meta-analysis done by Bocchino et al [11] has shown imaging abnormalities to be present at a pooled frequency of 43.5% at one-year follow-up.

We found that an increase in the mean CT severity score of parenchymal/atelectatic bands was associated with decreased FVC (*p*-value = 0.023), and an increase in the mean scores of reticulations/septal thickening and parenchymal/atelectatic bands was associated with increased dyspnea severity (*p*-value 0.012 and 0.045 respectively). This association between FVC and imaging patterns is consistent with the research done by Balbi et al [22] and González et al. [15] who found that the persistence of imaging abnormalities on chest CT was associated with dyspnea and deranged PFTs. Another study by Kumar et al [14] showed that consolidation and honeycombing were associated with dyspnea, while GGOs, traction bronchiectasis, and reticular pattern were associated with abnormal PFTs. However, they found no association between parenchymal/atelectatic bands and dyspnea or PFT.

While previous studies [5, 12, 15, 22, 23] have explored associations between imaging findings and clinical symptoms/PFTs, their correlations were broad, often limited to “versus “abnormal” or “versus “non-fibrotic” lungs (Table 6). Our study meticulously examined how individual imaging findings related to both the mMRC dyspnea scale and PFTs.

**TABLE 6:** Comparison of our current study with other post-COVID-19 studies evaluating imaging patterns in patients recovering from severe acute COVID-19.

Studies	Time of evaluation	Commonly observed imaging patterns	Association between increased dyspnea and normal vs. abnormal lung	Association between abnormal PFT and normal vs. abnormal lung	Other strengths
Current study	Six months after discharge	Reticulations, GGOs, parenchymal bands	Reticulations and parenchymal bands associated with dyspnea	Abnormal PFTs associated with parenchymal bands	Individual scoring system for each finding - more quantitative and reliable
Kartik K et al [14]	Baseline, 3 and 12 months	GGOs, consolidation	Consolidation and honeycombing were associated with breathlessness. Parenchymal bands, GGOs, reticular pattern and traction bronchiectasis were not associated with dyspnea.	Consolidation, GGOs, honeycombing and traction bronchiectasis were associated with lower FVC. GGO and traction bronchiectasis correlated with a reduction in ppDLCO and ppFVC, while reticular pattern correlated with a reduction in ppDLCO	Assessed association between imaging patterns and other respiratory symptoms such as cough and chest pain
Guinto E et al [5]	⩾Three months after COVID-19	GGOs, reticulations, bronchiectasis, consolidations	Abnormal pulmonary findings associated with dyspnea	No correlation	Age correlated with imaging abnormalities Being a woman negatively correlated with the presence of reticulations, bronchiectasis, and consolidations
Han and chen et al. [12]	Two years after recovery from COVID-19	GGOs, reticulations, atelectasis, bronchiectasis	Respiratory symptoms occurred more frequently in participants with ILAs.	Abnormal DLCO correlated with patients with ILAs	-
Balbi et al. [22]	105 days after symptom onset	GGOs, reticulations	Abnormal lung findings associated with dyspnea	Associated with abnormal pulmonary findings	-
Han and Fan et al. [23]	Six months after discharge	Complete radiological resolution or residual GGOs or interstitial thickening	Fibrotic lung changes associated with dyspnea more frequently than non-fibrotic lung changes	Fibrotic lung changes were associated with abnormal DLCO more frequently than non-fibrotic lung changes	Fibrotic lung changes correlated with older age, ARDS, longer hospital stay, noninvasive mechanical ventilation, and higher initial chest CT score
González et al. [15]	Three months after discharge	Reticulations, fibrotic lesions	Abnormal pulmonary findings associated with dyspnea	Associated with abnormal pulmonary findings	Lung changes associated with age, noninvasive mechanical ventilation

Abbreviation: ARDS, acute respiratory distress syndrome; DLCO, diffusion capacity of the lung for carbon monoxide; GGOs, ground glass opacities; ILAs, interstitial lung abnormalities; ppDLCO, percentage predicted DLCO, PFT, pulmonary function tests; RP, reticular pattern.

Furthermore, our study employed an individualized scoring system for each CT finding. This meticulous approach has been shown to improve both the precision and specificity of our observations, allowing for a better understanding of the intricate relationships between radiologic features and clinical parameters. Our findings reveal a statistically significant correlation between the presence of parenchymal/atelectatic bands and reduced forced vital capacity (FVC), a key measure of lung function. Additionally, both parenchymal/atelectatic bands and reticulation/septal thickening demonstrated a marked association with the mMRC dyspnea scale, which quantifies the severity of breathlessness. These observations suggest a direct link between specific radiographic patterns, pulmonary function impairment and the level of dyspnea. This deeper understanding, facilitated by our individualized scoring system, holds great promise for refining future diagnostic and prognostic strategies for patients recovering from COVID-19 ARDS.

Our study had several limitations. First, it was a single-center study with a small sample size. Second, chest CT scans and PFT results during acute illness were not available for comparison. Third, we only included patients with severe COVID-19 which may have led to statistical bias and overestimation of pulmonary findings.

## Conclusion

The most common CT findings observed in COVID-19 ARDS survivors with persistent respiratory problems at six-month follow-up were reticulations/septal thickening, GGOs, parenchymal bands, traction bronchiectasis, and consolidation. Notably, the imaging patterns of reticulations/septal thickening and parenchymal bands/atelectasis were significantly associated with increased scores on the mMRC dyspnea scale; while parenchymal bands/atelectasis also showed a statistically significant correlation with decreased FVC. However, longer follow-up studies are warranted to validate these findings and guide treatment decisions.

## Summary Table

### What Is Known About This Subject?

• Computed tomography (CT) of the chest is an invaluable tool for assessing lung changes in Long COVID subjects
• Persistent lung parenchymal abnormalities are seen on follow-up CT in one-third of hospitalized COVID-19 survivors
• Ground glass opacities (GGOs), septal thickening, parenchymal bands, consolidation, and traction bronchiectasis are common CT findings

### What Does This Paper Add?

• The relationship between clinical features, pulmonary function tests, and lung changes seen on chest CT at 6 months in COVID-19 ARDS survivors
• Reticulations/septal thickening and parenchymal/atelectatic bands showed a significant relationship with an increased mMRC dyspnea score
• Parenchymal/atelectatic bands also showed a statistically significant correlation with decreased forced vital capacity (FVC)

## Concluding Statement

This work represents an advance in biomedical science because it shows a statistically significant association between clinical parameters (dyspnea and FVC) and individual CT imaging patterns seen in COVID-19 ARDS survivors.

## Data Availability

The raw data supporting the conclusions of this article will be made available by the authors, without undue reservation.
